# Hexane-1,6-diaminium bis­[3,4,5,6-tetra­bromo-2-(meth­oxy­carbon­yl)benzoate] methanol disolvate

**DOI:** 10.1107/S1600536811035537

**Published:** 2011-09-14

**Authors:** Jian Li

**Affiliations:** a Department of Chemistry and Chemical Engineering, Weifang University, Weifang 261061, People’s Republic of China

## Abstract

In the title compound, C_6_H_18_N_2_^2+^·2C_9_H_3_Br_4_O_4_^−^·2CH_4_O, the carboxyl­ate and meth­oxy­carbonyl groups in one of the anions form dihedral angles of 71.9 (3) and 60.7 (3)°, respectively, with the aromatic ring while in the other anion these angles are 68.4 (3) and 56.8 (3)°, respectively. In the crystal, the constituent units are linked into a two-dimensional network parallel to the *ab* plane by N—H⋯O and O—H⋯O hydrogen bonds.

## Related literature

For related structures, see: Li (2011*a* ▶,*b* ▶,*c* ▶).
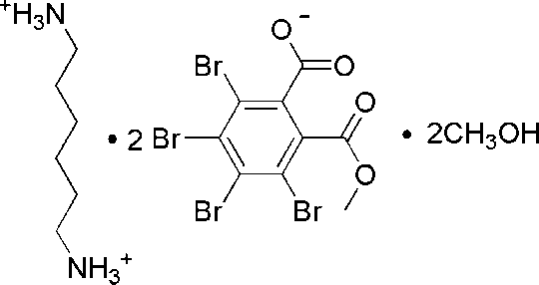


## Experimental

### 

#### Crystal data

C_6_H_18_N_2_^2+^·2C_9_H_3_Br_4_O_4_^−^·2CH_4_O
*M* *_r_* = 1171.74
Triclinic, 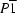

*a* = 8.1030 (6) Å
*b* = 13.5209 (12) Å
*c* = 17.7536 (17) Å
α = 89.388 (2)°
β = 83.744 (1)°
γ = 88.210 (2)°
*V* = 1932.5 (3) Å^3^
*Z* = 2
Mo *K*α radiation
μ = 8.35 mm^−1^
*T* = 298 K
0.27 × 0.26 × 0.25 mm

#### Data collection

Bruker SMART CCD area-detector diffractometer
Absorption correction: multi-scan (*SADABS*; Bruker, 1997 ▶) *T*_min_ = 0.211, *T*_max_ = 0.229
10205 measured reflections
6753 independent reflections
2917 reflections with *I* > 2σ(*I*)
*R*_int_ = 0.072

#### Refinement

*R*[*F* ^2^ > 2σ(*F* ^2^)] = 0.067
*wR*(*F* ^2^) = 0.158
*S* = 1.04
6753 reflections
421 parameters
H-atom parameters constrained
Δρ_max_ = 0.98 e Å^−3^
Δρ_min_ = −0.91 e Å^−3^

### 

Data collection: *SMART* (Bruker, 1997 ▶); cell refinement: *SAINT* (Bruker, 1997 ▶); data reduction: *SAINT*; program(s) used to solve structure: *SHELXS97* (Sheldrick, 2008 ▶); program(s) used to refine structure: *SHELXL97* (Sheldrick, 2008 ▶); molecular graphics: *SHELXTL* (Sheldrick, 2008 ▶) and *PLATON* (Spek, 2009 ▶); software used to prepare material for publication: *SHELXTL*.

## Supplementary Material

Crystal structure: contains datablock(s) global, I. DOI: 10.1107/S1600536811035537/ci5198sup1.cif

Structure factors: contains datablock(s) I. DOI: 10.1107/S1600536811035537/ci5198Isup2.hkl

Supplementary material file. DOI: 10.1107/S1600536811035537/ci5198Isup3.cml

Additional supplementary materials:  crystallographic information; 3D view; checkCIF report

## Figures and Tables

**Table 1:** Hydrogen-bond geometry (Å, °)

*D*—H⋯*A*	*D*—H	H⋯*A*	*D*⋯*A*	*D*—H⋯*A*
N1—H1*A*⋯O8^i^	0.89	2.01	2.890 (7)	171
N1—H1*B*⋯O10^ii^	0.89	1.99	2.840 (8)	158
N1—H1*C*⋯O4	0.89	2.02	2.892 (8)	167
N2—H2*A*⋯O4^iii^	0.89	2.00	2.877 (7)	168
N2—H2*B*⋯O9^iii^	0.89	2.04	2.885 (8)	159
N2—H2*C*⋯O8	0.89	1.99	2.859 (8)	165
O9—H9⋯O7^i^	0.82	1.96	2.743 (8)	160
O10—H10⋯O3	0.82	1.92	2.685 (8)	154

Symmetry codes: (i) 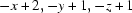; (ii) 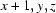; (iii) 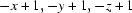.

**Fig. 1.**
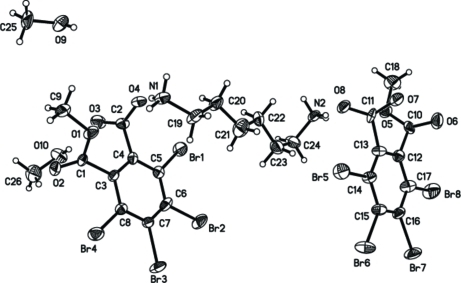
The molecular structure of (I), showing 30% probability displacement ellipsoids.

**Fig. 2.**
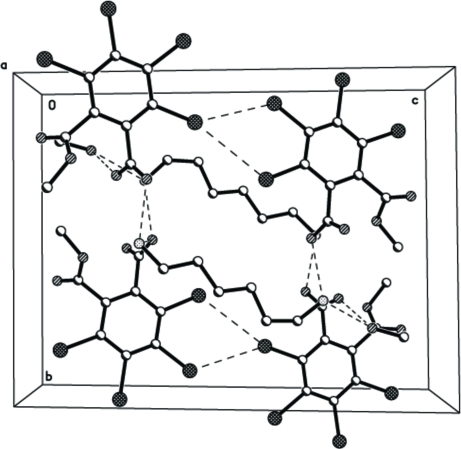
Part of the crystal structure of (I). Hydrogen bonds are shown as dashed lines.

## supplementary crystallographic information

### Comment

4,5,6,7-Tetrabromo-2-ethylisoindoline-1,3-dione is an important flame retardant. 3,4,5,6-Tetrabromo-2-(methoxycarbonyl)benzoic acid is an intermediate in the synthesis of this flame retardant. In this paper, the structure of the title compound, (I), is reported.

The asymmetric unit of (I) contains one hexane-1,6-diaminium cation, two 3,4,5,6-tetrabromo-2-(methoxycarbonyl)benzoate anions and two methanol solvent molecules (Fig. 1). The dihedral angles formed by the aromatic ring and the mean planes of the carboxylate and methoxycarbonyl groups are 71.9 (3)° and 60.7 (3)°, respectively, in one of the anions [with Br1], and 68.4 (3)° and 56.8 (3)°, respectively, in the other anion [with Br5]. The bond lengths and angles are in agreement with those observed for ethylammonium 2-(methoxycarbonyl)-3,4,5,6-tetrabromobenzoate methanol solvate (Li, 2011*a*), 2-methylanilinium 3,4,5,6-tetrabromo-2-(methoxycarbonyl)benzoate methanol monosolvate (Li, 2011*b*) and propan-1-aminium 3,4,5,6-tetrabromo-2-(methoxycarbonyl)benzoate *N*,*N*-dimethylformamide monosolvate (Li, 2011*c*). The crystal structure is stabilized by N—H···O and O—H···O hydrogen bonds (see Fig. 2 and Table 1).

### Experimental

A mixture of 4,5,6,7-tetrabromoisobenzofuran-1,3-dione (4.64 g, 0.01 mol) and methanol (15 ml) was refluxed for 30 min. Then hexane-1,6-diamine (0.58 g, 0.005 mol) was added to the above solution and mixed for 30 min at room temperature. After filtration, the solution was kept at room temperature for 5 d. Natural evaporation gave colourless single crystals of the title compound, suitable for X-ray analysis.

### Refinement

H atoms were initially located in a difference map and then refined in a riding model, with C–H = 0.96–0.97 Å, N–H = 0.89 Å, O–H = 0.82Å and *U*_iso_(H) = 1.2*U*_eq_(C) and 1.5*U*_eq_(O, N, methyl C).

### Crystal data

**Table:**

C_6_H_18_N_2_^2+^·2C_9_H_3_Br_4_O_4_^−^·2CH_4_O	*Z* = 2
*M**_r_* = 1171.74	*F*(000) = 1124
Triclinic, *P*1	*D*_x_ = 2.014 Mg m^−^^3^
Hall symbol: -P 1	Mo *K*α radiation, λ = 0.71073 Å
*a* = 8.1030 (6) Å	Cell parameters from 2038 reflections
*b* = 13.5209 (12) Å	θ = 2.3–22.4°
*c* = 17.7536 (17) Å	µ = 8.35 mm^−^^1^
α = 89.388 (2)°	*T* = 298 K
β = 83.744 (1)°	Block, colourless
γ = 88.210 (2)°	0.27 × 0.26 × 0.25 mm
*V* = 1932.5 (3) Å^3^	

### Data collection

**Table:**

Bruker SMART CCD area-detector diffractometer	6753 independent reflections
Radiation source: fine-focus sealed tube	2917 reflections with *I* > 2σ(*I*)
graphite	*R*_int_ = 0.072
φ and ω scans	θ_max_ = 25.0°, θ_min_ = 2.7°
Absorption correction: multi-scan (*SADABS*; Bruker, 1997)	*h* = −9→9
*T*_min_ = 0.211, *T*_max_ = 0.229	*k* = −14→16
10205 measured reflections	*l* = −21→18

### Refinement

**Table:**

Refinement on *F*^2^	Primary atom site location: structure-invariant direct methods
Least-squares matrix: full	Secondary atom site location: difference Fourier map
*R*[*F*^2^ > 2σ(*F*^2^)] = 0.067	Hydrogen site location: inferred from neighbouring sites
*wR*(*F*^2^) = 0.158	H-atom parameters constrained
*S* = 1.04	*w* = 1/[σ^2^(*F*_o_^2^) + (0.0483*P*)^2^] where *P* = (*F*_o_^2^ + 2*F*_c_^2^)/3
6753 reflections	(Δ/σ)_max_ = 0.001
421 parameters	Δρ_max_ = 0.98 e Å^−^^3^
0 restraints	Δρ_min_ = −0.91 e Å^−^^3^

### Special details

**Table:**

Geometry. All e.s.d.'s (except the e.s.d. in the dihedral angle between two l.s. planes) are estimated using the full covariance matrix. The cell e.s.d.'s are taken into account individually in the estimation of e.s.d.'s in distances, angles and torsion angles; correlations between e.s.d.'s in cell parameters are only used when they are defined by crystal symmetry. An approximate (isotropic) treatment of cell e.s.d.'s is used for estimating e.s.d.'s involving l.s. planes.
Refinement. Refinement of *F*^2^ against ALL reflections. The weighted *R*-factor *wR* and goodness of fit *S* are based on *F*^2^, conventional *R*-factors *R* are based on *F*, with *F* set to zero for negative *F*^2^. The threshold expression of *F*^2^ > σ(*F*^2^) is used only for calculating *R*-factors(gt) *etc*. and is not relevant to the choice of reflections for refinement. *R*-factors based on *F*^2^ are statistically about twice as large as those based on *F*, and *R*- factors based on ALL data will be even larger.

### Fractional atomic coordinates and isotropic or equivalent isotropic displacement parameters (Å^2^)

**Table:**

	*x*	*y*	*z*	*U*_iso_*/*U*_eq_	
Br1	0.31418 (12)	0.68365 (7)	0.41728 (5)	0.0652 (3)	
Br2	0.39769 (12)	0.92318 (6)	0.39517 (6)	0.0725 (3)	
Br3	0.56132 (12)	1.00591 (6)	0.22691 (6)	0.0648 (3)	
Br4	0.66925 (12)	0.84716 (6)	0.08825 (5)	0.0645 (3)	
Br5	1.15592 (12)	0.85386 (7)	0.58275 (5)	0.0659 (3)	
Br6	1.09419 (14)	1.09575 (7)	0.60733 (6)	0.0837 (4)	
Br7	0.94500 (13)	1.18227 (6)	0.77544 (7)	0.0795 (4)	
Br8	0.83327 (14)	1.02581 (7)	0.91531 (6)	0.0863 (4)	
N1	0.8619 (7)	0.5131 (4)	0.2862 (4)	0.051 (2)	
H1A	0.9149	0.4572	0.2974	0.077*	
H1B	0.9019	0.5339	0.2403	0.077*	
H1C	0.7540	0.5024	0.2869	0.077*	
N2	0.6252 (7)	0.6898 (4)	0.7155 (3)	0.048 (2)	
H2A	0.6007	0.6274	0.7072	0.073*	
H2B	0.5777	0.7084	0.7609	0.073*	
H2C	0.7349	0.6946	0.7138	0.073*	
O1	0.6599 (6)	0.5577 (4)	0.1436 (3)	0.0552 (17)	
O2	0.4895 (8)	0.6342 (4)	0.0704 (3)	0.070 (2)	
O3	0.2985 (7)	0.5302 (4)	0.2362 (4)	0.070 (2)	
O4	0.5040 (6)	0.5022 (3)	0.3069 (3)	0.0534 (18)	
O5	0.8163 (6)	0.7345 (4)	0.8560 (3)	0.0537 (17)	
O6	0.9820 (7)	0.8025 (4)	0.9332 (3)	0.075 (2)	
O7	1.1766 (7)	0.6946 (4)	0.7680 (3)	0.0629 (19)	
O8	0.9799 (6)	0.6778 (4)	0.6910 (3)	0.0542 (17)	
O9	0.5822 (6)	0.2186 (4)	0.1606 (3)	0.067 (2)	
H9	0.6672	0.2439	0.1713	0.080*	
O10	0.0507 (7)	0.6040 (4)	0.1616 (3)	0.072 (2)	
H10	0.1438	0.5914	0.1739	0.108*	
C1	0.5540 (9)	0.6306 (5)	0.1263 (4)	0.037 (2)	
C2	0.4161 (9)	0.5569 (5)	0.2704 (5)	0.045 (3)	
C3	0.5241 (9)	0.7015 (5)	0.1919 (4)	0.038 (2)	
C4	0.4548 (8)	0.6664 (5)	0.2630 (4)	0.038 (2)	
C5	0.4174 (8)	0.7333 (5)	0.3239 (4)	0.038 (2)	
C6	0.4523 (9)	0.8354 (6)	0.3122 (5)	0.051 (3)	
C7	0.5224 (9)	0.8691 (5)	0.2419 (4)	0.038 (2)	
C8	0.5601 (8)	0.8039 (5)	0.1809 (4)	0.039 (2)	
C9	0.6836 (11)	0.4745 (6)	0.0914 (5)	0.071 (3)	
H9A	0.5777	0.4547	0.0783	0.106*	
H9B	0.7371	0.4201	0.1152	0.106*	
H9C	0.7517	0.4940	0.0464	0.106*	
C10	0.9168 (10)	0.8012 (6)	0.8732 (5)	0.047 (3)	
C11	1.0597 (9)	0.7266 (6)	0.7303 (5)	0.042 (2)	
C12	0.9534 (9)	0.8749 (5)	0.8095 (5)	0.044 (2)	
C13	1.0192 (9)	0.8369 (5)	0.7381 (5)	0.045 (2)	
C14	1.0600 (9)	0.9033 (5)	0.6794 (5)	0.044 (2)	
C15	1.0332 (9)	1.0083 (6)	0.6896 (5)	0.051 (3)	
C16	0.9686 (9)	1.0448 (5)	0.7606 (5)	0.048 (3)	
C17	0.9298 (9)	0.9776 (6)	0.8195 (5)	0.053 (3)	
C18	0.7876 (11)	0.6499 (6)	0.9077 (5)	0.075 (3)	
H18A	0.8913	0.6266	0.9239	0.113*	
H18B	0.7392	0.5977	0.8821	0.113*	
H18C	0.7134	0.6699	0.9511	0.113*	
C19	0.8868 (11)	0.5894 (6)	0.3427 (5)	0.069 (3)	
H19A	0.8418	0.6523	0.3260	0.083*	
H19B	1.0051	0.5965	0.3443	0.083*	
C20	0.8082 (12)	0.5679 (6)	0.4215 (5)	0.071 (3)	
H20A	0.8753	0.5181	0.4449	0.086*	
H20B	0.6991	0.5414	0.4190	0.086*	
C21	0.7920 (12)	0.6598 (7)	0.4695 (5)	0.086 (4)	
H21A	0.9026	0.6811	0.4762	0.103*	
H21B	0.7386	0.7118	0.4420	0.103*	
C22	0.6969 (11)	0.6493 (6)	0.5457 (5)	0.057 (3)	
H22A	0.7596	0.6076	0.5778	0.069*	
H22B	0.5921	0.6184	0.5410	0.069*	
C23	0.6647 (11)	0.7515 (6)	0.5812 (5)	0.067 (3)	
H23A	0.7711	0.7801	0.5866	0.081*	
H23B	0.6098	0.7933	0.5462	0.081*	
C24	0.5626 (11)	0.7548 (6)	0.6564 (5)	0.069 (3)	
H24A	0.5567	0.8224	0.6745	0.082*	
H24B	0.4505	0.7362	0.6497	0.082*	
C25	0.6029 (12)	0.1902 (7)	0.0842 (5)	0.079 (3)	
H25A	0.6090	0.2481	0.0524	0.119*	
H25B	0.5102	0.1521	0.0736	0.119*	
H25C	0.7035	0.1509	0.0744	0.119*	
C26	0.0639 (12)	0.6378 (7)	0.0859 (5)	0.088 (4)	
H26A	−0.0277	0.6825	0.0790	0.132*	
H26B	0.0623	0.5825	0.0526	0.132*	
H26C	0.1662	0.6716	0.0745	0.132*	

### Atomic displacement parameters (Å^2^)

**Table:**

	*U*^11^	*U*^22^	*U*^33^	*U*^12^	*U*^13^	*U*^23^
Br1	0.0801 (6)	0.0632 (6)	0.0488 (6)	−0.0097 (5)	0.0118 (5)	−0.0028 (5)
Br2	0.0917 (7)	0.0543 (5)	0.0676 (7)	−0.0064 (5)	0.0128 (6)	−0.0280 (5)
Br3	0.0832 (6)	0.0289 (5)	0.0810 (8)	−0.0096 (4)	−0.0008 (6)	−0.0082 (4)
Br4	0.0886 (7)	0.0470 (5)	0.0542 (6)	−0.0124 (5)	0.0119 (6)	0.0021 (4)
Br5	0.0803 (6)	0.0675 (6)	0.0459 (6)	−0.0036 (5)	0.0112 (5)	−0.0003 (5)
Br6	0.1021 (8)	0.0542 (6)	0.0913 (8)	−0.0069 (6)	0.0031 (7)	0.0319 (5)
Br7	0.0937 (7)	0.0275 (5)	0.1189 (10)	0.0053 (5)	−0.0199 (7)	−0.0075 (5)
Br8	0.1153 (8)	0.0667 (6)	0.0716 (8)	0.0067 (6)	0.0141 (7)	−0.0274 (5)
N1	0.059 (4)	0.035 (4)	0.057 (5)	−0.004 (3)	0.007 (4)	0.000 (3)
N2	0.065 (4)	0.034 (4)	0.047 (5)	−0.006 (3)	−0.003 (4)	−0.009 (3)
O1	0.068 (4)	0.041 (3)	0.053 (4)	0.008 (3)	0.010 (3)	−0.017 (3)
O2	0.115 (5)	0.052 (3)	0.048 (4)	0.017 (3)	−0.027 (4)	−0.018 (3)
O3	0.065 (4)	0.058 (4)	0.091 (5)	−0.011 (3)	−0.031 (4)	0.016 (3)
O4	0.063 (4)	0.029 (3)	0.066 (4)	−0.004 (3)	0.003 (3)	0.003 (3)
O5	0.062 (3)	0.045 (3)	0.054 (4)	−0.019 (3)	−0.006 (3)	0.004 (3)
O6	0.099 (5)	0.082 (4)	0.050 (4)	−0.035 (4)	−0.022 (4)	0.009 (3)
O7	0.073 (4)	0.039 (3)	0.079 (5)	0.002 (3)	−0.018 (4)	−0.012 (3)
O8	0.069 (4)	0.039 (3)	0.054 (4)	−0.006 (3)	−0.005 (3)	−0.013 (3)
O9	0.066 (4)	0.060 (4)	0.074 (5)	−0.017 (3)	0.001 (4)	−0.017 (3)
O10	0.066 (4)	0.081 (4)	0.068 (5)	0.004 (3)	−0.001 (4)	0.022 (3)
C1	0.045 (5)	0.036 (4)	0.029 (5)	0.003 (4)	0.001 (4)	−0.014 (4)
C2	0.032 (5)	0.033 (5)	0.071 (7)	0.006 (4)	−0.014 (5)	0.002 (4)
C3	0.043 (5)	0.030 (4)	0.043 (5)	−0.001 (4)	−0.009 (4)	−0.007 (4)
C4	0.035 (4)	0.041 (4)	0.037 (5)	0.004 (4)	−0.006 (4)	−0.007 (4)
C5	0.020 (4)	0.046 (5)	0.048 (6)	0.002 (4)	−0.007 (4)	−0.002 (4)
C6	0.048 (5)	0.042 (5)	0.063 (6)	0.002 (4)	−0.010 (5)	−0.022 (4)
C7	0.039 (4)	0.031 (4)	0.043 (6)	−0.001 (4)	−0.007 (4)	−0.003 (4)
C8	0.033 (4)	0.036 (4)	0.047 (6)	0.001 (4)	−0.004 (4)	−0.003 (4)
C9	0.110 (7)	0.039 (5)	0.061 (7)	0.008 (5)	−0.004 (6)	−0.009 (5)
C10	0.068 (6)	0.053 (5)	0.022 (5)	0.010 (5)	−0.010 (5)	−0.003 (4)
C11	0.043 (5)	0.047 (5)	0.036 (5)	−0.014 (4)	−0.002 (4)	−0.018 (4)
C12	0.046 (5)	0.041 (5)	0.044 (6)	0.000 (4)	−0.004 (4)	−0.004 (4)
C13	0.048 (5)	0.029 (4)	0.054 (6)	0.003 (4)	0.003 (5)	0.008 (4)
C14	0.049 (5)	0.037 (5)	0.044 (6)	−0.003 (4)	0.008 (4)	−0.001 (4)
C15	0.051 (5)	0.039 (5)	0.061 (6)	−0.008 (4)	0.003 (5)	0.013 (4)
C16	0.043 (5)	0.031 (4)	0.070 (7)	0.003 (4)	−0.015 (5)	−0.011 (4)
C17	0.027 (4)	0.044 (5)	0.085 (7)	0.000 (4)	0.009 (5)	0.002 (5)
C18	0.084 (7)	0.075 (7)	0.064 (7)	−0.020 (6)	0.011 (6)	0.018 (5)
C19	0.095 (7)	0.045 (5)	0.064 (7)	−0.020 (5)	0.010 (6)	−0.019 (5)
C20	0.129 (8)	0.047 (5)	0.036 (6)	−0.027 (5)	0.007 (6)	−0.006 (4)
C21	0.096 (7)	0.091 (7)	0.068 (8)	−0.012 (6)	0.007 (6)	−0.023 (6)
C22	0.073 (6)	0.065 (6)	0.036 (6)	−0.004 (5)	−0.016 (5)	−0.002 (4)
C23	0.086 (7)	0.063 (6)	0.052 (7)	0.015 (5)	−0.015 (6)	−0.002 (5)
C24	0.094 (7)	0.056 (6)	0.054 (7)	0.019 (5)	−0.006 (6)	0.007 (5)
C25	0.121 (8)	0.086 (7)	0.029 (6)	−0.009 (6)	0.008 (6)	−0.016 (5)
C26	0.099 (8)	0.095 (8)	0.065 (8)	0.019 (6)	0.014 (7)	−0.017 (6)

### Geometric parameters (Å, °)

**Table:**

Br1—C5	1.900 (7)	C9—H9A	0.96
Br2—C6	1.907 (8)	C9—H9B	0.96
Br3—C7	1.897 (7)	C9—H9C	0.96
Br4—C8	1.878 (8)	C10—C12	1.510 (11)
Br5—C14	1.923 (8)	C11—C13	1.522 (10)
Br6—C15	1.902 (8)	C12—C17	1.406 (10)
Br7—C16	1.881 (7)	C12—C13	1.417 (10)
Br8—C17	1.906 (9)	C13—C14	1.387 (10)
N1—C19	1.480 (10)	C14—C15	1.439 (10)
N1—H1A	0.89	C15—C16	1.401 (11)
N1—H1B	0.89	C16—C17	1.394 (11)
N1—H1C	0.89	C18—H18A	0.96
N2—C24	1.482 (10)	C18—H18B	0.96
N2—H2A	0.89	C18—H18C	0.96
N2—H2B	0.89	C19—C20	1.502 (11)
N2—H2C	0.89	C19—H19A	0.97
O1—C1	1.342 (8)	C19—H19B	0.97
O1—C9	1.460 (9)	C20—C21	1.509 (12)
O2—C1	1.170 (9)	C20—H20A	0.97
O3—C2	1.247 (9)	C20—H20B	0.97
O4—C2	1.238 (9)	C21—C22	1.489 (11)
O5—C10	1.294 (9)	C21—H21A	0.97
O5—C18	1.468 (10)	C21—H21B	0.97
O6—C10	1.241 (9)	C22—C23	1.529 (11)
O7—C11	1.281 (9)	C22—H22A	0.97
O8—C11	1.214 (9)	C22—H22B	0.97
O9—C25	1.404 (9)	C23—C24	1.494 (11)
O9—H9	0.82	C23—H23A	0.97
O10—C26	1.409 (11)	C23—H23B	0.97
O10—H10	0.82	C24—H24A	0.97
C1—C3	1.510 (10)	C24—H24B	0.97
C2—C4	1.525 (10)	C25—H25A	0.96
C3—C4	1.408 (10)	C25—H25B	0.96
C3—C8	1.432 (10)	C25—H25C	0.96
C4—C5	1.418 (10)	C26—H26A	0.96
C5—C6	1.426 (10)	C26—H26B	0.96
C6—C7	1.392 (11)	C26—H26C	0.96
C7—C8	1.404 (10)		
			
C19—N1—H1A	109.5	C16—C15—Br6	120.9 (6)
C19—N1—H1B	109.5	C14—C15—Br6	119.5 (6)
H1A—N1—H1B	109.5	C17—C16—C15	118.6 (7)
C19—N1—H1C	109.5	C17—C16—Br7	121.7 (6)
H1A—N1—H1C	109.5	C15—C16—Br7	119.6 (6)
H1B—N1—H1C	109.5	C16—C17—C12	122.0 (8)
C24—N2—H2A	109.5	C16—C17—Br8	119.1 (6)
C24—N2—H2B	109.5	C12—C17—Br8	118.9 (6)
H2A—N2—H2B	109.5	O5—C18—H18A	109.5
C24—N2—H2C	109.5	O5—C18—H18B	109.5
H2A—N2—H2C	109.5	H18A—C18—H18B	109.5
H2B—N2—H2C	109.5	O5—C18—H18C	109.5
C1—O1—C9	116.6 (6)	H18A—C18—H18C	109.5
C10—O5—C18	118.2 (7)	H18B—C18—H18C	109.5
C25—O9—H9	109.5	N1—C19—C20	114.6 (7)
C26—O10—H10	109.5	N1—C19—H19A	108.6
O2—C1—O1	124.2 (7)	C20—C19—H19A	108.6
O2—C1—C3	126.4 (7)	N1—C19—H19B	108.6
O1—C1—C3	109.2 (7)	C20—C19—H19B	108.6
O4—C2—O3	125.8 (7)	H19A—C19—H19B	107.6
O4—C2—C4	118.7 (7)	C19—C20—C21	111.4 (7)
O3—C2—C4	115.4 (7)	C19—C20—H20A	109.3
C4—C3—C8	120.5 (7)	C21—C20—H20A	109.3
C4—C3—C1	119.3 (6)	C19—C20—H20B	109.3
C8—C3—C1	120.1 (7)	C21—C20—H20B	109.3
C3—C4—C5	119.8 (7)	H20A—C20—H20B	108.0
C3—C4—C2	117.8 (6)	C22—C21—C20	115.7 (8)
C5—C4—C2	122.3 (7)	C22—C21—H21A	108.4
C4—C5—C6	119.2 (7)	C20—C21—H21A	108.4
C4—C5—Br1	118.3 (5)	C22—C21—H21B	108.4
C6—C5—Br1	122.5 (6)	C20—C21—H21B	108.4
C7—C6—C5	120.5 (7)	H21A—C21—H21B	107.4
C7—C6—Br2	121.6 (6)	C21—C22—C23	109.3 (7)
C5—C6—Br2	117.9 (6)	C21—C22—H22A	109.8
C6—C7—C8	121.1 (7)	C23—C22—H22A	109.8
C6—C7—Br3	119.6 (5)	C21—C22—H22B	109.8
C8—C7—Br3	119.3 (6)	C23—C22—H22B	109.8
C7—C8—C3	118.9 (7)	H22A—C22—H22B	108.3
C7—C8—Br4	121.2 (5)	C24—C23—C22	116.2 (7)
C3—C8—Br4	119.8 (5)	C24—C23—H23A	108.2
O1—C9—H9A	109.5	C22—C23—H23A	108.2
O1—C9—H9B	109.5	C24—C23—H23B	108.2
H9A—C9—H9B	109.5	C22—C23—H23B	108.2
O1—C9—H9C	109.5	H23A—C23—H23B	107.4
H9A—C9—H9C	109.5	N2—C24—C23	114.9 (7)
H9B—C9—H9C	109.5	N2—C24—H24A	108.5
O6—C10—O5	124.5 (7)	C23—C24—H24A	108.5
O6—C10—C12	123.5 (8)	N2—C24—H24B	108.5
O5—C10—C12	111.9 (7)	C23—C24—H24B	108.5
O8—C11—O7	126.5 (7)	H24A—C24—H24B	107.5
O8—C11—C13	118.8 (7)	O9—C25—H25A	109.5
O7—C11—C13	114.7 (7)	O9—C25—H25B	109.5
C17—C12—C13	120.0 (7)	H25A—C25—H25B	109.5
C17—C12—C10	122.7 (7)	O9—C25—H25C	109.5
C13—C12—C10	117.3 (6)	H25A—C25—H25C	109.5
C14—C13—C12	118.4 (7)	H25B—C25—H25C	109.5
C14—C13—C11	122.0 (7)	O10—C26—H26A	109.5
C12—C13—C11	119.2 (7)	O10—C26—H26B	109.5
C13—C14—C15	121.4 (7)	H26A—C26—H26B	109.5
C13—C14—Br5	119.2 (5)	O10—C26—H26C	109.5
C15—C14—Br5	119.4 (6)	H26A—C26—H26C	109.5
C16—C15—C14	119.6 (7)	H26B—C26—H26C	109.5
			
C9—O1—C1—O2	−5.1 (11)	O5—C10—C12—C17	125.7 (8)
C9—O1—C1—C3	171.0 (6)	O6—C10—C12—C13	120.5 (9)
O2—C1—C3—C4	115.6 (9)	O5—C10—C12—C13	−56.4 (9)
O1—C1—C3—C4	−60.4 (9)	C17—C12—C13—C14	0.4 (12)
O2—C1—C3—C8	−62.0 (11)	C10—C12—C13—C14	−177.5 (7)
O1—C1—C3—C8	122.0 (7)	C17—C12—C13—C11	174.1 (7)
C8—C3—C4—C5	1.2 (11)	C10—C12—C13—C11	−3.9 (11)
C1—C3—C4—C5	−176.4 (7)	O8—C11—C13—C14	−72.6 (10)
C8—C3—C4—C2	178.7 (7)	O7—C11—C13—C14	108.3 (9)
C1—C3—C4—C2	1.1 (10)	O8—C11—C13—C12	114.0 (9)
O4—C2—C4—C3	108.4 (9)	O7—C11—C13—C12	−65.1 (9)
O3—C2—C4—C3	−69.7 (9)	C12—C13—C14—C15	−1.5 (12)
O4—C2—C4—C5	−74.1 (10)	C11—C13—C14—C15	−174.9 (7)
O3—C2—C4—C5	107.8 (9)	C12—C13—C14—Br5	178.1 (6)
C3—C4—C5—C6	−0.6 (11)	C11—C13—C14—Br5	4.7 (11)
C2—C4—C5—C6	−178.0 (7)	C13—C14—C15—C16	1.7 (12)
C3—C4—C5—Br1	177.0 (5)	Br5—C14—C15—C16	−177.8 (6)
C2—C4—C5—Br1	−0.4 (9)	C13—C14—C15—Br6	179.2 (6)
C4—C5—C6—C7	−0.1 (11)	Br5—C14—C15—Br6	−0.3 (9)
Br1—C5—C6—C7	−177.6 (6)	C14—C15—C16—C17	−0.9 (12)
C4—C5—C6—Br2	179.3 (5)	Br6—C15—C16—C17	−178.4 (6)
Br1—C5—C6—Br2	1.8 (9)	C14—C15—C16—Br7	176.5 (6)
C5—C6—C7—C8	0.2 (12)	Br6—C15—C16—Br7	−1.0 (9)
Br2—C6—C7—C8	−179.2 (6)	C15—C16—C17—C12	−0.1 (12)
C5—C6—C7—Br3	178.1 (5)	Br7—C16—C17—C12	−177.4 (6)
Br2—C6—C7—Br3	−1.3 (9)	C15—C16—C17—Br8	−177.4 (6)
C6—C7—C8—C3	0.4 (11)	Br7—C16—C17—Br8	5.3 (9)
Br3—C7—C8—C3	−177.5 (5)	C13—C12—C17—C16	0.4 (12)
C6—C7—C8—Br4	−175.4 (6)	C10—C12—C17—C16	178.2 (8)
Br3—C7—C8—Br4	6.7 (9)	C13—C12—C17—Br8	177.7 (6)
C4—C3—C8—C7	−1.1 (11)	C10—C12—C17—Br8	−4.5 (10)
C1—C3—C8—C7	176.5 (7)	N1—C19—C20—C21	−162.4 (8)
C4—C3—C8—Br4	174.8 (6)	C19—C20—C21—C22	172.8 (8)
C1—C3—C8—Br4	−7.6 (10)	C20—C21—C22—C23	−169.9 (8)
C18—O5—C10—O6	−5.4 (11)	C21—C22—C23—C24	177.0 (8)
C18—O5—C10—C12	171.5 (6)	C22—C23—C24—N2	54.7 (11)
O6—C10—C12—C17	−57.4 (11)		

### Hydrogen-bond geometry (Å, °)

**Table:**

*D*—H···*A*	*D*—H	H···*A*	*D*···*A*	*D*—H···*A*
N1—H1A···O8^i^	0.89	2.01	2.890 (7)	171
N1—H1B···O10^ii^	0.89	1.99	2.840 (8)	158
N1—H1C···O4	0.89	2.02	2.892 (8)	167
N2—H2A···O4^iii^	0.89	2.00	2.877 (7)	168
N2—H2B···O9^iii^	0.89	2.04	2.885 (8)	159
N2—H2C···O8	0.89	1.99	2.859 (8)	165
O9—H9···O7^i^	0.82	1.96	2.743 (8)	160
O10—H10···O3	0.82	1.92	2.685 (8)	154

Symmetry codes: (i) −*x*+2, −*y*+1, −*z*+1; (ii) *x*+1, *y*, *z*; (iii) −*x*+1, −*y*+1, −*z*+1.
